# Text memorization: An effective strategy to improve Chinese EFL learners’ argumentative writing proficiency

**DOI:** 10.3389/fpsyg.2023.1126194

**Published:** 2023-04-04

**Authors:** Qunfeng Wang

**Affiliations:** School of Humanities and Foreign Languages, Xi’an University of Technology, Xi’an, China

**Keywords:** English writing proficiency, text memorization strategy, text memorization process, language learning strategies, EFL learners

## Abstract

This study aims to explore the impact of text memorization strategies on Chinese EFL learners’ English argumentative writing proficiency, the process of their text memorization, and specific strategies deployed for the enhancement of the memorization effect. Seven text memorization tests, one pre-test, and one post-test were administrated to 33 Chinese English majors to, respectively, examine students’ memorization outcomes as well as their English argumentative writing proficiency before and after memorizing seven model English writings. Data were also collected through interviews with the 12 top scorers in text memorization tests. The results showed that text memorization as a foreign language learning strategy significantly impacted the improvement of EFL learners’ argumentative writing proficiency. Moreover, in the text memorization process, in which varieties of strategies were employed, it was found that storage was preceded by understanding among the majority of the interviewees. Since text memorization was found to be advantageous to EFL learners’ writing proficiency, a new system of text memorization strategies was developed in the current study to provide both scholars and teachers with insight into text memorization strategies associated with the writing skills of EFL learners.

## 1. Introduction

The relationship between language learning strategies (LLSs) and language achievements has long been the subject of research, much of which suggests that LLSs, as an aid, are effective in successful language learning (Griffiths and Soruç, 2020). Since some of the strategies seem to remain inordinately attached to or associated with language skills in specific areas (Oxford, 2017), there is increasing interest in the investigation into the strategies employed in language skill areas, such as reading and writing. Among the four modalities: listening, reading, speaking, and writing in foreign language learning (FLL), writing, at times, frustrates and challenges the majority of foreign language learners (Fareed et al., 2016). The development of writing skills requires English as a foreign language (EFL) learners to present clear ideas in line with their thinking by the application of linguistic knowledge, for instance, the arrangement of words, clauses, and sentences in a coherent manner based on systematic rules (Hyland, 2003). For EFL learners, memorizing this knowledge by using effective memorization strategies is the initial step to not only develop their writing skills but also improve their overall language proficiency. Therefore, memorization is viewed as one of the essential learning strategies for FLL, the importance of which is highlighted in the definition of learning strategies. It refers to operations that learners deploy for acquisition, storage, retrieval, or use of information (Dansereau, 1985), or intentional behavior and thoughts used by learners during learning to better help them understand, learn, or remember new information (Richards and Platt, 1992).

Given the significance of memorization in language learning, due attention has been given to revealing the relationship between the choice of language learners’ memorization strategies and their outcome of vocabulary learning. Previous research suggests a positive correlation between memorization strategy use and vocabulary achievement (Rashidi and Omid, 2011). Since memory strategies are frequently applied to memorize vocabulary and structures during the early phase of language learning, studies on memorization thus far seem to be limited to linking memorization with just the amelioration of learning vocabulary, the smaller units of language. However, in many Asian nations, for example, China, the memorization material has extended from a single word or character to complex texts, the larger units of language, such as sentences, paragraphs, and whole essays. When acquiring L1 from kindergarten to university, Chinese students are instructed and encouraged to memorize varieties of texts. The content and style of these texts vary from Chinese proverbs and poems to full articles by representatives of masters of Chinese literature. As text memorization has sustained and developed into a traditional Chinese literacy learning strategy, it has also been transferred to the process of FLL and become a crucial approach for both Chinese acquisition and English learning.

On account of the wide employment of memorization as a learning strategy, Chinese learners used to be stereotypically characterized in Western educational settings, as passive learners who rely heavily on memorization of material in their learning process (Chan and Rao, 2009), but much more recent research has shed light on the vital roles that memorization plays in FLL (Mouziraji and Mouziraji, 2015; Khamees, 2016; Sonmez, 2018). A body of research into Chinese EFL learners’ memorization strategy use indicated that Chinese learners were not rote memorizers but active archivers who applied a series of memorization strategies to facilitate their language learning (Biggs, 1996; Kember, 2000; Li and Cutting, 2011). However, in many previous studies, “text memorization” was not differentiated from “memorization” academically. In other words, the two terms were mostly used interchangeably without consideration of the difference in the length of the material for memorization, which would influence the specific memorization strategy use and memorization process. Based on the differentiation of text memorization from memorization, a few studies attempted to unfold the relationship between text memorization and the language proficiency of EFL learners. It was suggested that text memorization worked effectively to enhance EFL learners’ language competence (Dai and Ding, 2010). Few studies have been conducted to relate text memorization as a learning strategy to proficiency in English writing, with a systematic observation and analysis of strategies involved in the text memorization process.

In China, though Chinese EFL learners employ text memorization strategies widely in their English learning processes, many of them use a variety of specific text memorization strategies unsystematically since, until now, there is no system for text memorization strategy available to refer to and help enhance the effects of text memorization. Moreover, in the field of teaching English as a foreign language, the focus of English writing teaching lies in writing strategies, such as planning strategy, while-writing strategy, and revising strategy [as suggested by Petri and Czárl (2003)] or other relevant writing skills, for example, idea development. As such, the influences that the traditional Chinese literacy learning strategy (text memorization), may exert on Chinese ESL learners’ English writing outcomes have been ignored. Therefore, with the aim of providing EFL learners and teachers with new insight into the use of text memorization strategies and its impact on EFL learners’ English writing proficiency, this study tends to focus on uncovering the relationship between text memorization and English writing proficiency, particularly the proficiency of English argumentative writing, through the exploration of Chinese EFL learners’ processes of text memorization and the specific strategies involved.

## 2. Literature review

### 2.1. Classifications of LLSs and memorization strategies

Since language learning requires skillful employment of an array of strategies, efforts have been made in the previous studies to classify LLSs with diverse perspectives to present a system of LLSs. The classification scheme developed by Rubin (1981) includes two general categories. The first group of strategies contributes directly to learning and is subdivided into clarification/verification, monitoring, memorization, guessing/inductive inferencing, deductive reasoning, and practice. The second group of strategies contributes indirectly to learning (e.g., creating opportunities for practice and production tricks). According to O’Malley and Chamot (1990), LLSs are composed of cognitive strategies, metacognitive strategies, social strategies, and affective strategies. However, Oxford (1990) depicted them as direct strategies that include memory strategies, cognitive strategies, and compensation strategies, and others as indirect strategies composed of metacognitive strategies, social strategies, and affective strategies. Through the consideration of the overlap between cognitive strategies and metacognitive strategies, Macaro (2001) presented the categories of strategies along a continuum of subconscious (or less conscious), based on which, LLSs are divided into direct strategies at one end and conscious and indirect strategies at the other. Cohen (2014) classified LLSs in a new light on the basis of the reasons for using the strategies, for example, strategies for language learning vs. language use, strategies by language skill area, and strategies according to function (namely, metacognitive, cognitive, affective, or social). Despite the different classifications, the recognition of systematic LLSs not only enables scholars and teachers to examine the learning strategies but also helps language learners control their learning and become more proficient in language learning.

As well as LLSs having been classified by some, scholars have also pointed out the important roles that memorization strategy plays in FLL. Therefore, attempts have been made to identify the specific memorization strategies and categorize them to present a system of memorization to help EFL learners improve their learning outcomes. Memorization, one of the direct learning strategies that Rubin (1981) classified, includes four subsets: take notes of new items, pronounce out loud, find an association, and use other mechanical devices. Oxford (1990) grouped memory strategy into four categories: creating mental links, applying images and sound, reviewing well, and employing action, and subdivided them into 10 specific strategies. The strategy for formally committing the materials that are not acquired naturally through exposure to memory is included in the language learning strategies identified by Cohen (2014). Since memorizing or storing the elements of a new foreign language is the foundation for enlarging learners’ knowledge and paving the way for FLL, it is recognized as the initial key to FLL. However, more recent studies on the classification of memorization can rarely be found. To enable language learners to be aware of and discover the specific memorization strategies that suit them best in their language learning, more studies on memorization classifications need to be conducted.

### 2.2. Memorization, understanding, and rote learning

Rote learning is traditionally defined as memorization based on repetition without understanding. Therefore, once rote learning or memorization is adopted, learners are described as rote memorizers, passive and unproductive rote learners, or low-level strategy users. In particular, language learners with Asian backgrounds such as Chinese, Japanese, and Korean have long been viewed and labeled as rote memorizers (Mok et al., 2001; Mathias et al., 2013), which means they are inclined to include memorization as a major strategy for language learning. This concept of the connections between memorization and rote learning is common mainly in the West, and it prompted Marton et al. (1996) to put forward the “paradox” that heavy reliance on memorization makes Chinese learners successful language learning achievers, which is later explained by the findings of the studies that classify and redefine memorization. Based on the learners’ intention to understand or the lack thereof, Mugler and Landbeck (2000) identified two meanings of memorization: rote learning, which implies a lack of understanding, and memorization, which implies understanding. Moreover, in terms of sequential order between the two processes, three patterns were found: understanding, then memorization; memorization, then understanding; and a combination of both. A similar exploration into memorization revealed three memorization molds associated with understanding (Marton et al., 2005): rote memorization, in which memorization precedes understanding (Hess and Azuma, 1991); meaningful memorization 1, in which memorization succeeds understanding (Kember and Gow, 1990); and meaningful memorization 2, in which memorization and understanding are seen as simultaneous and combined in the learning process (Kember, 1996). Thus far, it has been proven by the previous documents that the employment of a memorization strategy does not necessarily mean that language learners learn only in a mechanical way when understanding is engaged in the learning procedure. Therefore, memorization strategy, as one of the indispensable parts of FLLs, needs to be considered and interpreted in a different light.

### 2.3. Vocabulary memorization and text memorization

Previous research is primarily concerned with two types of memorization strategies according to the length of material to memorize: strategies to memorize smaller linguistic units of language, which is termed “vocabulary memorization,” and strategies to memorize larger linguistic units of language (e.g., complex material of a consecutive text, including sentences, paragraphs, and full essays), which is termed “text memorization.” Much research is focused on the former, and a substantial body of studies has been conducted to explore a range of strategies employed by EFL learners to memorize vocabulary (Oxford, 1990; Klapper, 2008; Al-Qaysi and Shabdin, 2016). Furthermore, a number of memorization strategies have been designed purposely and suggested for the instruction of EFL learners on vocabulary learning (Abbasi et al., 2018; Badr and Abu-Ayyash, 2019). As many scholars (Schmitt, 1997; Takač, 2008; Sinhaneti and Kyaw, 2012) have found, a large group of subsets of memory strategies identified so far, for example, memory strategies by Oxford (1990), are effective vocabulary memory strategies, which are believed to be closely associated with only vocabulary learning and have become part of vocabulary learning strategies. Therefore, when memorization as a language learning strategy was accounted for in many previous studies, memorization was generally used to refer to strategies applied by language learners to commit vocabulary to memory, with little consideration of text memorization.

Though text memorization is widely accepted by EFL learners with Asian cultural backgrounds as an efficacious language learning strategy, little research has been conducted on it. A few studies have revealed that text memorization facilitates FLL in many respects, such as vocabulary, grammar, structure, and language skills. Based on an interview with three winners of national English-speaking competitions or debate tournaments in China, Ding (2007) reported that the practice of text memorization facilitates FLL through the enhancement of noticing and rehearsal because collocations and sequences can be learned and then borrowed for productive use. Moreover, the habit of tending to details of language in the context of language input was developed. This conclusion was echoed by another study (Dai and Ding, 2010), which found that text memorization exerts a positive influence on EFL learners’ language proficiency and writing performance due to the accuracy and variation of formulaic sequences used. Through collecting data from a group of Chinese learners and teachers (*N* = 62) from 15 middle schools and universities, Yu (2013) concluded that the employment of text memorization not only contributes to the improvement of learning of vocabulary, phrases, sentence structures, grammar, and language skills such as writing and speaking but also affords psychological satisfaction, which is built on EFL learners’ sense of achievement and confidence. Compared with the interview-based studies that have clarified the manifold benefits that EFL learners gained from text memorization, more recent research by Harris (2015) proposed a systematic pattern for the memorization of a story or dialog, which is advantageous to language learning, known as a top-down and bottom-up pattern. The top-down mold, requiring examination of the overall content of the text, consists of three steps: start by understanding the main idea of the entire text, then break the text down into manageable sections to understand the main idea of each, and finally, analyze each sentence for the general content and main idea. The bottom-up mold involves careful analysis of language elements at the lexical level and how the meaning was created by the combination of words.

In summary, previous literature has suggested that text memorization as an effective foreign language learning strategy has a positive impact on EFL learners’ overall English proficiency (i.e., Ding, 2007; Dai and Ding, 2010; Yu, 2013), but few studies have been conducted to reveal the relationship between text memorization and EFL learners’ writing proficiency. Furthermore, it has been revealed in previous studies that understanding, which is involved in the process of memorization, makes EFL learners with Asian cultural backgrounds active learners rather than rote memorizers. However, little is known about how text memorization proceeds when understanding is engaged in the process of text memorization. In addition, the majority of existing research is centered on the strategies of vocabulary memorization, and few studies have attempted to present a system for text memorization strategies that EFL learners apply in their processes of memorizing complex and consecutive text. Therefore, the findings of this study fill the gap by answering three questions:

1. Does text memorization as a foreign language learning strategy affect the improvement of EFL learners’ argumentative writing proficiency?
2. When Chinese EFL learners employ text memorization strategies, is understanding involved in their memorization process? If yes, what is the text memorization process of these successful memorizers?
3. What specific strategies do the successful Chinese memorizers who learn English as a foreign language employ to memorize the texts, and how could these strategies be classified to formulate a system for text memorization?

## 3. Method

### 3.1. Design of the study

Mixed-method research was conducted, in which both quantitative and qualitative methods were used. In the majority of educational settings, random assignment of students is hardly possible, thus, quasi-experiments are often conducted to create the comparisons, from which treatment-caused change is inferred (Zoltán, 2007). Therefore, in the present study, quasi-experiments were designed to answer the first question about whether text memorization could help improve Chinese EFL learners’ writing proficiency with the application of quantitative research methods. The experimental sample involved one intact university class. A pre-test that required this class group to produce argumentative writing was implemented to assess their writing proficiency before the treatment. Afterward, this group of students was given a text to memorize each week (seven in total) and was required to report the full text in a memorization test in the following week; thus, their text memorization effects were examined by a total of seven memorization tests. Finally, a post-test was also administrated to this group of students, requiring them to produce another piece of argumentative writing. The post-test was targeted at evaluating their writing proficiency after the treatment. Then a comparison between students’ writing performances in the pre-test and post-test was made to discover whether students’ writing proficiency was improved after the treatment. To answer the second and third questions, the qualitative research method was adopted. After all the tests were completed, semi-structured interviews were conducted with the 12 students who performed best in the text memorization tests to gain information about these successful memorizers’ text memorization processes and the specific text memorization strategies involved, based on which, a system for text memorization was attempted to be formulated.

### 3.2. Participants

Convenience sampling was applied to include one intact class group that consisted of 33 students as participants (29 women and four men) who were in their second year of studies at one of the universities in China. The participants were accepted as English majors by the university through the national college entrance examination and were between 19 and 20 years of age. The author had taught them *Intensive Reading* for more than half a year. When the research was conducted, the participants had been learning English as their major for approximately one and a half years by taking a variety of professional English courses designed for college English majors, such as *Intensive Reading*, *Extensive Reading*, and *Grammar*. This meant that the participants were on the way to becoming qualified English majors. All the participants were informed of the purpose of the study and agreed to participate in the study, but they were free to withdraw at any time. At the time of data analysis, all the participants (*n* = 33) were included.

### 3.3. Instruments

#### 3.3.1. Text memorization tasks and text memorization tests

Seven argumentative writings were selected as model writings (refer to Appendix A) for students to memorize. Two university English teachers were invited and, together with the author, examined the quality of seven selected writings by discussion and reached a consensus that they could be used as model writings. To select model writings, two major aspects were concerned: the quality of the writings and the topics of the writings. The criteria to ensure the high quality of the model writings lay in such aspects as lexical resources, grammatical structures, accuracy, coherence and cohesion, and idea development. The topics of the writings were to be diverse and closely associated with life experiences that students are familiar with. The topics of the finally selected seven model writings ranged from technology and life (model writing 1), campus life (model writings 2, 5, 7, and 8), and family issues (model writing 3), to environmental issues (model writing 4) and social issues (model writing 6). Students were given 1 week to memorize a model writing, and then a memorization test was conducted. Students’ text memorization quality was examined by requiring them to recall and verbatim write out the texts of the model writings without referring to the model writings and without the use of dictionaries. In total, seven memorization tests were administrated within 7 weeks, one for each week.

#### 3.3.2. Pre-test and post-test

In the pre-test, the writing task (see Appendix C) from the 2017 Test for English Majors-Band 4 (TEM 4) was used to assess the participants’ writing proficiency before the task of memorizing the seven model writings was undertaken. In the post-test, the one from 2019 (refer to Appendix C) was used for the evaluation of students’ writing performance after the memorization work was done. There were two reasons why the two writing tasks of TEM 4 were used in the present study. First, TEM 4 was a written test designed by the National Education Committee of People’s Republic of China and used to assess the English proficiency of Chinese English majors in their second-year studies at the university since 1991. The students who participated in the present study were all sophomores majoring in English. Therefore, the previous writing tasks of TEM 4 were appropriate to be used to evaluate the participants’ writing proficiency. Second, the topics of the writing tasks of TEM 4 covered education, university life, society, etc., which Chinese college students are familiar with. This would minimize the negative influences that the lack of topic knowledge may have exerted on the students’ writing performances.

#### 3.3.3. Semi-structured interview

By using both qualitative and quantitative approaches, the best of both paradigms (quantitative and qualitative research) could be deployed (Zoltán, 2007). Many researchers have developed qualitative studies as the support and supplement for quantitative research to explore EFL learners’ learning behavior, for instance, the use of LLSs (He and Shi, 2008; Jiang and Smith, 2009; Yu, 2017). In view of research questions 2 and 3, interviews with participants about their experiences of text memorization were used as a logical approach in the present research to triangulate the quantitative data in a broad sense. The top 12 students in the score list for the memorization of seven model English argumentative writing tests were chosen to be interviewed. The interviews were semi-structured with two major interview guide questions (refer to Appendix E) developed by the author with reference to the interview guild questions in the study by Jiang and Smith (2009), which were aimed to detect Chinese learners’ strategies used in English learning. The first interview question of the present study was focused on the participants’ text memorization processes of the model writings and the specific text memorization strategies involved. The second question was aimed at collecting information about students’ attitudes toward text memorization strategies. The reliability of the interview data was enhanced in four ways. First, all the interviewees were students whom the author had taught for more than half a year; therefore, a good relationship established between them enabled the interview to proceed in a more natural and conversational manner. Second, to ensure that participants had enough time to recall their memorization experiences and provide authentic information about their memorization procedures, the students were informed of the interview questions 1 day before the interview. Third, sub-questions such as “*What learning strategies did you use to help you commit the model writings to memory?”* and “*In what aspects do you think text memorization is effective to improve your English argumentative writing?”* were designed to encourage and assist students in providing more details about their memorization process. Fourth, the individual interviews were conducted in Chinese instead of English so that the students could express their ideas more clearly in their native language. The interviews were conducted with flexibility, and a group of interview techniques suggested by Zoltán (2007), such as carry-on feedback and encouraging elaboration, were employed by the author. For instance, when one interviewee reported, “I’m able to read the text fluently,” the interviewer raised the probe question or follow-up question, “*Do you mean you need to read it out loud or not?”* to both confirm the intended meaning of the utterance and increase the richness and depth of the response. In addition, the wording of the prepared questions was sometimes geared to better suit the different personalities of the interviewees or make the interviews more natural.

### 3.4. Data collection and analysis procedures

Before the study, all the participants were informed of the purpose of the study, the roles that participants play in data collection, and the confidential and voluntary nature of the research. On the Monday of the first week of the research, a pre-test was conducted, and model writing 1 was given to students to memorize. On the Monday of the second week, memorization test 1 was administrated, and model writing 2 was handed out. On the Monday of the third week, memorization test 2 was administrated, and model writing 3 was handed out. Such a process was repeated so that within 8 weeks, seven text memorization tests had been conducted. In the ninth week, after all the memorization tests had been completed, the post-test was administrated. Two experienced university English teachers were given 2 weeks (weeks 10 and 11) to rate students’ performances in all memorization and writing tests. The evaluation criterion (see Appendix B) for memorization tests was code-signed by them to make the rating consistent. The reliability of the two raters was acceptable according to the Pearson correlation coefficient (test 1, r = 0.91; test 2, r = 0.86; test 3, r = 0.90; test 4, r = 0.88; test 5, r = 0.91; test 6, r = 0.92; test 7, r = 0.88). Thus, the average score achieved by each student was used as the final score, which indicates each student’s memorization effect of seven model argumentative writings. To ensure the reliability of the pre-test and post-test, the scoring criterion (see Appendix D) was also implemented by both two university English teachers. The interrater reliability was acceptable for the present study according to the Pearson correlation coefficient (pre-test, r = 0.67; post-test, r = 0.64). Therefore, the average score given by the two raters to each student was used as the final score, representing each student’s writing proficiency. Then, descriptive statistics, such as means and standard deviations of the participants’ writing proficiency before and after completion of the memorization tasks, were reported and interpreted. The paired samples *t*-test was adopted to examine whether participants’ writing proficiency was improved after the text memorization assignments were accomplished. Pearson correlation coefficients were used to represent the relationships between participants’ performance in the memorization of seven model writings and their writing proficiency after the text memorization tasks were completed.

The semi-structured interviews were conducted in the office of the English department in the ninth week by the author after all the experimental tests had been completed. Each participant agreed to the interview being recorded by smartphone for data analysis for the study. All the interviews were then transcribed and translated into English by the author. The transcribed and translated texts were double-checked, during which the author sought validation from the interviewees, through smartphone conversations, when ambiguity arose. Then the translated texts were analyzed to gain information about the participants’ text memorization processes (including whether understanding was involved) and the specific text memorization strategies used to enhance their text memorization effects. More importantly, based on the analysis, varieties of specific text memorization strategies were classified to formulate a system for text memorization. In the process of analysis, to ensure the accurate interpretation of the interviewees’ viewpoints, exchanges through smartphone or WeChat voice calls were conducted for confirmation when necessary.

## 4. Results and findings

### 4.1. Comparison of students’ writing performances between pre-test and post-test

Descriptive statistics of the pre-test and post-test shown in Table 1 demonstrate that the mean scores of the post-test, M = 12.21, SD = 0.48, are improved compared to those of the pre-test (M = 10.64, SD = 0.54). The results of the paired-samples *T*-test suggest that there is a statistically significant difference between the mean scores of pre-test and post-test, *t*(32) = −3.88, *p* = 0.00 < 0.05, indicating that students’ argumentative writing proficiency was greatly improved after the memorization tasks of seven English argumentative writings had been completed.

**Table 1:** Paired-samples *T*-test for pre-test and post-test.

	N	M	SD	T	DF	Sig.
Pre-test	33	10.64	0.54	−3.88	32	0.00
Post-test	33	12.21	0.48			

### 4.2. Relationship between memorization effect of seven model writings and writing performances after the completion of text memorization tasks

The Pearson correlation coefficients in Table 2 show a significant positive relationship between participants’ performances in text memorization of seven model writings and their writing proficiency after the completion of the text memorization tasks, r = 0 0.63, *p* < 0.01, which indicates that the better the achievements made in text memorization of seven model writings, the higher the proficiency in English argumentative writing.

**Table 2:** Pearson correlation coefficients for text memorization test of seven model writings and post-test.

	Memorization test	Post-test
Memorization test	1	
Post-test	0.63^**^	1

** *p* < 0.01, two-tailed.

### 4.3. Strategies used for memorization of the model writings and students’ perspectives

#### 4.3.1. Strategies used for understanding that facilitates memorization

All the interviewees except for interviewees 2 and 10 emphasized the importance of understanding before they memorized the texts, but the strategies used to understand were reported to be differential. Two of them (1 and 8) mentioned that translation of the whole text into Chinese was done prior to memorization to help them understand the content and main ideas of the writings. Judging from the tone of their utterances, the claim of such a way for memorization seemed awkward to them, presumably due to it being time-consuming or other unknown reasons. However, it was a personal and satisfying approach that they appreciated.

Another practice that many of the interviewees, including interviewees 3, 4, 5, 8, 11, and 12, developed was to read through the texts to get the main idea of the model writings. Afterward, they would seek assistance from the dictionary if the words and phrases became barriers preventing them from understanding the texts. “On Wednesday morning, when I have no classes from 10 am to 12 pm [*sic*], I will read through the text and look up the words I do not know in the dictionary. The initial things for memorization like that.” After recalling her first step, the use of a dictionary for memorization of the model writings, interviewee 4 continued to state that she did not favor learning by rote. Underlining the primary significance of understanding with strong opposition to rote memorization, she said, “It is not good to start to memorize without understanding the texts, for example, mechanical memorization or rote memorization.” Similarly, interviewee 5 attempted to understand the text when the memorization task started, but unlike interviewees 1 and 8, the frequent method used for understanding was not translating but repeated reading. “I do not intend to translate the texts. When I read them over and over, I can understand the ideas that the texts convey,” she said.

Compared with the majority of the interviewees’ notions that understanding precedes memorization, interviewee 10 reported that she adopted mechanical memorization by two mechanical means: storing the words’ locations and typing the model writings. She explained:

“My approaches to memorizing the texts are a bit mechanical. I have a unique method. Unlike others, I often memorize the specific location of a word on the writing handed to me, and it can be stored in my brain. When I forget the content, I can recall where the word is located. There is another good method, which seems a bit time-consuming. I type the whole model writing using the computer. This method helps me memorize it faster, but it is also easier to forget because I don’t fully understand it.”

#### 4.3.2. Memorizing through text analysis

Among the strategies adopted, another frequently used approach was to analyze the structure of complicated sentences to facilitate memorization with the grammatical knowledge acquired. The normal practice that the majority of these successful memorizers deployed, not excluding interviewees 1, 3, 4, 6, 7, 9, and 11, was to identify the subject, the predicate, and the object of a long sentence that was formed with more linguistic elements, such as phrases and clauses. This process is, in fact, the analysis of the hierarchal order of a sentence, which was conducted with intention of breaking down complicated sentences into smaller units to ease the process of memorization. By being aware of the subject (either a noun, a noun phrase, or a nominal clause) and the predicate (a verb or a verb phrase), the efforts made resulted in the linguistic portrayal of a fundamental tree diagram. However, as second-year students, they were not aware of that tree diagram since they had not yet attended any courses relevant to linguistics or syntax. The conspicuous objective of such analysis, as they explained, was to make their memorization tasks easier.

“I often analyze the main structure of the sentences. A seemingly long sentence would not be complicated anymore if the subject, the predicate, and the object are [sic] detected … The rest part [sic] of a sentence would be modifiers like adjectives. This is the way I do to [sic] simplify a complicated sentence” (interviewee 1).

In addition to sentence structure analysis, another skill that interviewees 6 and 3 mentioned was writing an outline for each selected writing, which was firmly believed to offer intelligible guidance to them to recall the contents and opinions of the writings. Their inceptive attention was attached to the organization of the writing and the ways by which the ideas developed. In other words, when the entire stages of memorization were examined, analysis and storage of the outline always predated that of the language details. Interviewee 3 outlined the given model writing by clarifying the main ideas, analyzing the supporting details, and picking up on the keywords. She acknowledged that such a method worked well for the memorization of the whole essay. The importance of outlining the writings was echoed by interviewee 6, describing how an outline is produced:

“The awareness of the organization of the whole writing makes the memorization tasks easier. An introduction of the topic discussed is often made in the beginning, which is followed by one argument of the topic. Then the author’s thesis statement is put forward. In the main body, the core notion is often analyzed from two respects. Finally, the conclusion is made. My focus is always on the keywords and linking words like however, therefore, etc., so that an outline of the writing can be drawn. I memorize them based on the outline afterward.”

#### 4.3.3. Storage strategies for memorization

Recitation, a widely accepted L1 learning strategy in China to increase the effect of memorization, was often employed by the interviewees. Seven of them, interviewees 1, 2, 4, 5, 8, 9, and 11, reported that reciting the texts was one of the necessary channels that assisted them in committing the texts to memory. Two forms of recitation were reported: audibly (loudly or in a low voice) and silently, according to the places where they recited the texts. Choosing this memorization strategy was a personal preference depending on individual learning styles and characteristics.

To be able to memorize the whole essay, interviewees 1 and 2 developed the habits of reading and repeatedly reciting aloud, the value of which was emphasized by interviewee 2, who said, “Compared with reading and reciting silently, the memorization effect through reading and reciting loudly and repeatedly is much better.” However, in addition to reading and reciting loudly, reading and reciting softly was also acceptable to interviewee 5 because reading and reciting by heart did not enhance their memorization. Such an audible approach to reading and reciting, either loudly or softly, was shared by interviewee 9, who emphasized that when in the library, she would retrieve the texts from her mind and write them out instead of uttering any sound. Interviewee 4 said that she read and recited silently in the library since audible reading and recitation would disturb others and were not allowed. However, she added, “If the learning environment permits, I often read as loudly as what I have done in my middle school during the time for morning reading,” (a period of time, approximately 20–30 min during the morning of each school day for middle school students to read either Chinese or English texts loudly in China).

The depictions of the interviewees’ recitation procedures revealed that reading the texts, either aloud or in a low voice, was a commonly used approach that was incorporated into the process of their recitation. Students intended to read out so that they could hear the contents of the texts, thus enhancing the memorization effect. Followed by audible reading, reciting to themselves audibly or silently reinforced their memorization. The unavoidable forgetfulness urged students to refer to the texts either by reading loudly or silently and then the texts would be recited again. Such a reading–reciting cycle would be repeated several times until they were conscious that the texts had been memorized. Therefore, repetition was taken as one of the indispensable strategies for strengthening the memorization effect. Moreover, reviewing on a daily basis or at intervals of several days was verbally reported by interviewees 1, 2, 3, and 5.

#### 4.3.4. Students’ perspectives on the impact of text memorization on English writing proficiency

All the students interviewed valued the impact of memorizing the model writings on improving their writing performances. The majority stressed that the benefits gained from it were extremely positive. The enhancements can be illustrated in three aspects of argumentative writing. The most conspicuous effect highlighted by interviewees 1, 2, 3, 4, 5, 7, and 12 was that memorization of the model writings enabled them to acquire the ability to construct a piece of English argumentative writing and develop the ideas in a coherent manner. For instance, interviewee 1 claimed that the regular structure of the argumentative writing and thinking patterns were learned as they accomplished the memorization assignments. Another advancement was that the students learned diversified sentence patterns and extended their vocabulary. For example, interviewees 4 and 12 revealed that their previous argumentative writings were all completed using the translation mold; that is, a Chinese sentence was generated in their minds first and was then translated into a simple English sentence. More often than not, the sentence was organized with improper diction and grammar that caused ambiguity and confusion. With more model writings memorized, this translation mold used for English writing was gradually abandoned on the grounds that native-like sentences could be produced with the application of more advanced words, correct collocations, and complicated sentence patterns.

## 5. Discussion

### 5.1. Impact of text memorization on EFL learners’ argumentative writing proficiency

The present study examines the impact of text memorization as a foreign language learning strategy on the improvement of EFL students’ argumentative writing proficiency. The results revealed that text memorization of the model essays is significantly effective in enhancing the argumentative writing proficiency of Chinese EFL learners, which is consistent with the previous finding (Dai and Ding, 2010) that the adoption of text memorization in FLL contributes to greater progress in EFL learners’ writing ability. At the lexical level, the memorization of full text enables EFL learners to store a string of words that are formed in a fixed sequence, termed “formulaic sequences” (Schmitt, 2004), thus reducing the possibility of EFL learners making grammatical errors (Boers and Lindstromberg, 2008). If the knowledge of FSs is absent, EFL learners depend on their L1 knowledge to possibly combine irrelevant words to generate incorrect collocations in English argumentative writing. As a result, negative transfer occurs with the production of deviant L2 combinations (Ellis, 2008). However, with the accumulation of these prefabricated patterns stored in the mind through text memorization, students are able to retrieve such chunks or multi-word units easily and put them to use, with no need to arrange them through word selection and grammatical sequencing (Tremblay and Baayen, 2010). In terms of the syntactical and textual level, when learners memorize whole texts with the help of a series of text memorization strategies, especially the syntactical and textual analysis strategy, the relevant sentence structures and the arrangements of the argumentative essays can be retrieved and, more importantly, imitated by EFL learners to produce better writings of their own. This finding is supported by previous studies, suggesting that with the recognition of how information in texts is packaged and organized through text analysis, more cohesive and coherent writings can be composed (Tovar, 2016). Text analysis is not intended to encourage copying but rather to promote awareness of style and writing subskills. In short, in the process of text memorization, both small and large linguistic forms are noticed by EFL learners and stored in their short-term memory. When successive text memorization helps to transfer short-term memory into long-term memory, there is a greater likelihood that EFL learners can retrieve the retained information or knowledge related to the lexicon, syntax, and textual organization to generate better-structured English sentences. Therefore, their arguments on a variety of the given topics can be clearly presented for communication in the form of writing. In other words, it is the memory-based system that enables EFL learners to access and deploy chunks of language, on which language fluency depends (Skehan, 1995).

### 5.2. Text memorization process of Chinese EFL learners

**Figure 1:**
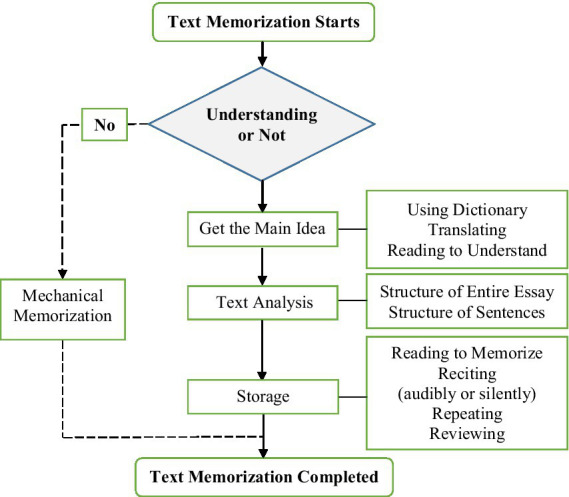
Diagram of text memorization process and strategies used by Chinese EFL learners (source: original).

When it comes to memorization of larger linguistic units of a foreign language, i.e., full texts, the current study showed that Chinese EFL learners’ memorization process is more complex than memorization merely by repetition, as it has previously been described. The memorization process, including memorization strategies, is presented in Figure 1. When memorization starts, mechanical memorization is possibly adopted in the absence of understanding. However, if understanding is involved, such strategies as using a dictionary, translating, and reading are used for comprehension of the model writings first. Then the structures of the whole writing and sentences are analyzed to facilitate memorization. Afterward, audible or silent reading and reciting are repeated by language learners to store the texts. In this process, the reviewing strategy helps learners return to the procedure of understanding to deepen their comprehension, which further strengthens memorization.

In the present study, two types of memorization practices were identified for storing and recalling the texts reliably and retrieving them easily. The less common was memorizing mechanically without the engagement of understanding, whereas the most common, which was also discovered in the studies of Hess and Azuma (1991) and Kember and Gow (1990), was giving priority to understanding, followed by memorization. The third type, the integration or combination of memorization and understanding explored by Mugler and Landbeck (2000) and Marton et al. (2005), was not reported by the participants in this study, though the author assumed that it existed. With regard to understanding, it was found that the most frequent and effective strategy employed by Chinese EFL learners was a top-down model. This mold shares similarities with the top-down approach to facilitating text memorization in the study of Harris (2015), which reported three steps for text memorization: (1) understanding the main idea of the entire text, (2) analyzing the structure of the full text, and (3) analyzing the structure of sentences. Since the top-down approach and the bottom-up approach could complement each other (Davies, 1988), the bottom-up mold based on the careful breakdown of sentences with a focus on lexical and syntactic forms was also found in this study. Following this pattern, EFL learners can be informed of the formal and content schemata for the construction of the essay on the top level, as well as the lexis and syntactic forms that realize it at the sentence level.

### 5.3. Text memorization strategies by Chinese EFL learners

**Figure 2:**
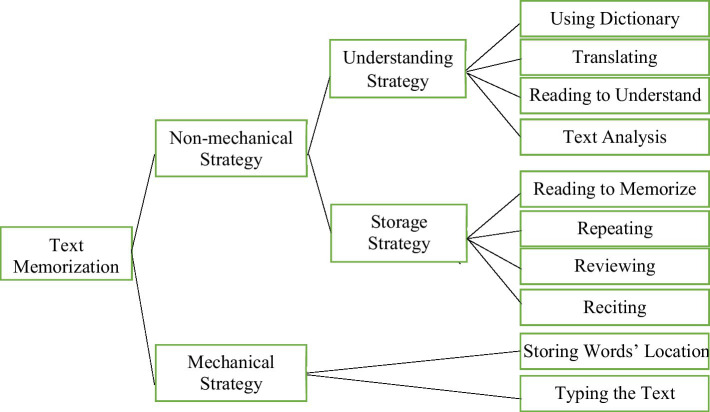
Diagram of text memorization strategies used by Chinese EFL learners (source: original).

The present study found that in the process of the memorization of the given model writings, all of the successful Chinese memorizers actively employed a range of strategies to improve their memorization effect, which were concluded as the text memorization strategies. These strategies worked together to contribute to the storage, retention, recall, or retrieval of the texts that Chinese EFL learners tended to memorize. A new strategy for text memorization used by Chinese EFL learners was developed based on the findings of the present study and is shown in Figure 2.

Considering whether understanding is involved or not, text memorization strategies can be divided into two major classes: mechanical strategies and non-mechanical strategies. The term, mechanical strategies, is preferred to generally describe one of the intentional approaches of text memorization. The evidence obtained in the current study indicates that Chinese EFL learners do not just rely on repeating, they also seek out other strategies, such as keeping in mind the word’s location in the writing and typing the text on a computer. Though these strategies are believed to be mechanical, they are different from conventional rote memorization, referring to memorization by mere repetition in the previous study (Lazaric, 2012). Therefore, two groups: storing the word’s location and typing the text, were discovered and grouped into mechanical strategies. The non-mechanical strategies consist of understanding strategies for text comprehension that facilitate memorization and storage strategies for remembering and retrieval of the texts. Understanding strategies fall into four categories: using a dictionary, translating, reading to understand, and text analysis including structural analysis of the entire writing and structural analysis of the sentence. Storage strategies are made up of four other categories: reading to memorize, reciting (audibly or silently), repeating, and reviewing. It should be noted that two forms of reading strategies were identified according to the goals that Chinese EFL learners tend to reach. The connotation of “reading to understand” is the same as that of “reading” in the phrase “reading a novel,” which means to comprehend the contents. However, “reading to memorize” is defined as the strategy of reading the texts audibly or silently with the intention of retaining the texts in memory.

There is no consensus on how strategies are defined and categorized (Takač, 2008), thus, classification conflicts do exist. This study suggests that translating and analyzing are two strategies for understanding that fall under the text memorization strategy. However, both of these types were considered cognitive strategies in the study of Oxford (1990). Similarly, repeating, a cognitive strategy in Oxford’s taxonomy of strategies, is viewed as a set of the text memorization strategy in this study. Such different classifications could be explained in two respects: (1) Previously, memorization strategies were investigated as the major strategies for memorizing vocabulary, the smaller units of language. Therefore, a shift in concern from memorization of vocabulary to memorization of full texts leads to the distinct classifications of specific memorization strategies. (2) The roles that a strategy plays in different learning phases impact the divisions of the strategies. For example, when translating is seen as a tool for manipulation or transformation of the target language by the learner, it is recognized as a cognitive strategy. However, in the present study, translating is identified as a sub-strategy of text memorization because it is deployed for the purpose of storage. It is understandable that when the role of a certain strategy changes in a given task in language learning, it can be sorted into two groups simultaneously, which are meant to overlap (Oxford, 1990). More recently, emphasizing the free and flexible operations of LLSs, Oxford (2017) proposed that while a strategy might be thought of as being in a certain group, other potential functions of that strategy should also be seriously considered. Despite the unclear boundaries between the categories of some strategies, the system of text memorization strategies developed in the current study could still present a wide-ranging framework to both examine text memorization strategy that many Chinese EFL learners accepted as a means to underpin their text memorization outcome and extend our understanding of it.

## 6. Conclusion and limitations

The current study contributes to the identification of the relationship between LLS use and language proficiency, particularly the relationship between the use of text memorization as a foreign language learning strategy and English argumentative writing proficiency by presenting further evidence from a Chinese FLL context. The results suggest a significantly positive relationship between text memorization and Chinese EFL learners’ writing outcomes. It is concluded that text memorization as a foreign language learning strategy is effective in improving EFL learners’ writing proficiency, which previous literature on language learning strategy use in general settings and specific language skill areas has seldom documented. Moreover, this study is the first attempt to explore and develop a new system of text memorization strategies based on the analysis of the text memorization processes and strategies deployed by Chinese EFL learners. The suggestion on the differentiation of memorization of larger units of language, such as the full text of an essay, from memorization of smaller units of language, such as vocabulary, provides new insight into memorization strategy research within the field of LLSs.

Although this study detected a variety of strategies used for text memorization, which have been proven to work effectively in improving Chinese EFL learners’ writing proficiency, other strategies to increase the text memorization effect are likely to offer a more comprehensive interpretation of Chinese EFL learners’ text memorization strategy use. Future studies should focus on the discovery of these strategies and how these strategies are involved in the process of Chinese EFL learners’ text memorization processes. Moreover, a multitude of independent variables potentially impact strategy use (Grainger, 2012), thus leading to differential language performance. Gender, in this study, could have played a role because 29 out of 33 subjects were women. In addition, this study was conducted in China, with Chinese EFL learners as the objects, so when EFL learners’ cultural backgrounds or socio-ecological contexts change, whether the strategy used and the impact of text memorization on EFL learners’ writing proficiency would differ is anticipated to be uncovered in future studies.

## Data availability statement

The original contributions presented in the study are included in the article/Supplementary material, further inquiries can be directed to the corresponding author.

## Author contributions

The author confirms being the sole contributor of this work and has approved it for publication.

## Funding

This study was supported by the 2019 Social Science Foundation of Shaanxi Province, China (2019M006).

## Conflict of interest

The author declares that the research was conducted in the absence of any commercial or financial relationships that could be construed as a potential conflict of interest.

## Publisher’s note

All claims expressed in this article are solely those of the authors and do not necessarily represent those of their affiliated organizations, or those of the publisher, the editors and the reviewers. Any product that may be evaluated in this article, or claim that may be made by its manufacturer, is not guaranteed or endorsed by the publisher.
