# Supplementary material for: Text memorization: An effective strategy to improve Chinese EFL learners’ argumentative writing proficiency
Source: Front Psychol. 2023 Apr 4;14:1126194. doi: 10.3389/fpsyg.2023.1126194 (PMC10112396; doi:10.3389/fpsyg.2023.1126194)
Supplement: Supplementary file 1 [file Data_Sheet_1.docx]

**Appendix A: Seven Model Writings**

**Some contend that modern technology facilities our life, but others worried that high-tech products influenced us negatively. Please state your opinions on it.**

**Model writing 1**

Nowadays, high-tech products like smart-phone, iPad etc. have been exceedingly popular among the masses, which has stirred up a fierce debate among the specialists, not excluding the economists and the sociologists. However, the drawbacks brought about by it to people, especially the youngsters, are serious and manifold.

Among the various downsides, what concerns people most is that the inventions of modern technology, which are being applied in different walks of life, seem to be cutting us off from contact with our fellow human beings. Family members are isolated when each individual plays on the phone in his own room, so the opportunities to share his happiness and miseries are noticeably reduced. Friends, peers and intimates keep a certain social distance by being indulged in network-related activities, for instance, online games and shopping. Another serious consequence lies in that less contacts and communications possibly result in a sense of loneliness, and even worse, cause the psychological issue. Although short or accidental loneliness does not contribute to the mental diseases, long-term separation can cause certain emotional disorders, thus exerting negative influences on people’s life quality.

Modern technology, seemingly, is helping human beings conduct personal activities more easily and independently, but in fact, it is setting human beings apart and posing a threat to the human health. Therefore, immediate actions should be taken to eliminate the unfavorable impacts resulted from modern technology.

**Please give comments on whether college students should join in the Students’ Union actively when they pursue academic achievements.**

**Model writing 2**

In the recent past, a heated debate on whether college students should actively become a member of Students’ Union has been the concern for a number of people, including parents, educators and even sociologists. Some of them assert that spending time in being involved in various school activities would reduce the time on academic pursuits. However, as a member of the Sports Department of the Students’ Union, I, firmly, contend that the benefits brought by joining in it are conspicuously observed.

The immediate benefit, which cannot be ignored, is that the university societies provide the opportunities for college students to make improvements on their abilities to deal with people with different backgrounds and personalities. Therefore, in the process of being involved in the routine works assigned by the Students’ Union, one’s communication skills could be enhanced to a large extent. Another noticeable benefit is that by being exposed to the formal or informal occasions, the knowledge acquired is endless. For instance, being a member of Yoga club and taking the relevant courses, one may acquire the approaches to keep fitness as well as the information regarding the psychological health.

To conclude, it is strongly recommended that while pursing academic achievements, college students join in the different departments of the Students’ Union actively for their personal developments. To achieve the goal, making the youngsters be aware of the positive impacts of health would be the initial step. Moreover, both school administrators and teachers are expected to appeal to these adolescents to apply for the membership in line with their interests.

**In many countries today, people in cities either live alone or in small family units, rather than in large, extended family groups. Is this a positive or negative trend?**

**Model writing 3**

It is true that cities are seeing a rise in smaller families and one-person household while the extended family is becoming a rarity. In my opinion, this is a negative development.

As families become smaller, the traditional family unit is disappearing, which could exert a negative impact on children as they grow up. In a nuclear family or single-parent household, childcare becomes an expensive and stressful part of daily life. Without the help of grandparents or aunts and uncles, busy parents must rely on babysitters, or after-school clubs to take care of younger children. The absence of adult family members can mean that television and the Internet become the primary influences on children’s behavior. It is no surprise that the decline of the extended family has been linked to a rise in psychological and behavioral problems amongst the teenagers.

The trend towards living alone is even more damaging. Individuals who live on their own have nobody to talk to in person, so they cannot share problems or discuss the highs and lows of daily life. The lack of human contact at home is necessarily replaced by passive distractions, such as video games, online chat rooms or Internet surfing. This type of existence is associated with boredom, loneliness, and feelings of isolation, all of which are factors that are known to the increased risk of mental illness.

In conclusion, I believe that individuals thrive when they are part of larger family groups, and so it is worrying that many people are choosing to live alone or in such small family units.

**Tourism has boosted the economy and provide the opportunity for people to see the world. However, with the constructions of the hotels and other business conducts, it has caused a lot of environmental problems. Do you agree with it?**

**Model writing 4**

Tourism, an indispensable part of modern business, has long been taken as being effective to boost economy. However, a number of environmentalists contend that such commercial conducts would pose a threat to the earth where humans lived for thousands of years. Indeed, the drawbacks brought about by it to mankind are grievous and manifold.

Among the various downsides, what concerns people most is that the ecological system could be damaged if too many business activities in regard to tourism are conducted. In fact, countless animals, including some rare species, are facing the danger of losing homes when human beings dominate their lands. Another negative influence is that various commercial attempts could cause water contamination, thus endangering the health of human beings. A latest report, released by a scientific research center, reveals that in the past 20 years, with the destruction of the environment the number of the new deceases is in an increase, so health problems worry people a lot.

The urgent problem now is to make people recognize the importance of the issue. What need to be considered is complex, but for the sake of better our future living condition and health, we should not only encourage more effective ways to finance the environmental bodies, but also establish relevant educational system to instruct people to love the planet that we are living on.

**As a matter of fact, humans, including college students, will have to be confronted with burdens from life and study. How will you deal with the problems caused by stress in life?**

**Model writing 5**

Nowadays, a large amount of factors, such as academic pursuits, interpersonal communication and job hunting etc. contribute to the stress that college students are being confronted with. As such spiritual tension may lead to psychological and health issues, some effective methods could assist the adolescents to stay recovered by eliminating the pressure.

As it turns out, the immediate approach adopted by the youth to tackle the various problems caused by stress is to share the concerns, worries and nervousness with their best friends, peers or intimates. By conducting a face to face conversation or talking through the phone, or Internet, there is a great possibility that the stress could be examined from a positive perspective. Another productive means, which cannot be neglected, is that regular exercises like taking a daily brisk walk or doing some sports will be conducive to stress-reduction. While being engaged in such physical activities, one could be relaxed and free his mind out of the mental burden.

In order to tackle the problem, constant efforts should be made and relevant measures should be taken. On the one hand, educational institutions and mass media should take the responsibility to arouse people’s awareness on the consequences caused by the unavoidable stress. On the other hand, it is suggested that each individual form a proper life outlook, taking the pressure as a part of the life. As a result, stress will not be a factor that discourages people from leading a pleasant life.

**In recent years, the excessive use of packages has become quite a serious problem in China What’s your attitude towards excessive packaging?**

**Model writing 6**

In the recent past, a great number of goods with excessive packaging are surprisingly sold in stores and supermarkets, which has caused a wide concern among the masses, including the economists and sociologists. Indeed, several reasons can account for such issue.

The immediate reason advanced is that in order to earn satisfactory profits, more and more products with splendid packaging are produced. The desire to explore the markets can be understandable, but such an attempt to waste natural resources on making the packaging is disgraceful. Another clear justification, which cannot be neglected, is that upon the influences of “face culture”, the beautifully decorated commodities seem to enjoy great popularity. With the fast development of economy, the income of Chinese people has been gradually increased. As a result, they have more money to purchase the expensive products with excessive packaging, which is mostly considered as the symbol of social status.

For the sake of the preservation of the natural resources and the formation of a positive life outlook, constant efforts should be made and relevant measures should be taken to tackle the problem of excessive packaging. On the one hand, governments and mass media should take the responsibility to arouse people’s awareness on the consequences caused by excessive packaging by spreading abundant information. On the other hand, stricter rules, regulations, and even laws could be enacted to restrict the manufacturers from using natural resources for excessive packaging.

**It is recently reported that six students who shared a dorm at a local university hired a cleaner to do laundry and cleaning once a week. And each of them paid her 60 yuan a month. Do you think whether college students should hire cleaners?**

**Model writing 7**

It was recently reported that a few college students hired a help to do some cleaning in their dorm, which has been the concern for a number of educators, not excluding university administrators and the sociologists. These students attributed this decision to the limited time that should be spent on the academic pursuits. As a matter of fact, this chain of reasoning is seriously flawed when the value of university life is taken into consideration.

The most noticeable justification why seeking a cleaner to do the laundry is not advocated is that university education involves not only knowledge exploration but also character building. To manage to deal with the daily routines and live independently are one of the crucial objectives for all the adolescents, let alone the ones who receive the higher education. There is no doubt that the ethos to complete the tasks of one’s own should be evoked and developed during this critical period of lifetime. Another clichéd- but-true answer to the opposition to such an inappropriate conduct lies in that one of the opportunities to cultivate the teamwork spirit is deprived if a cleaner is employed. By working together, each takes his responsibility to build a cosy home and the friendship among the roommates would be significantly enhanced with the awareness of the importance of the good of the cooperation.

Therefore, it is safe to come to the conclusion that college students should learn to stand on their own feet by seeing to their chores. Hiring a cleaner is definitely not a wise choice expected to make.

**Appendix B: Criterion for evaluation of students’ memorization effect of model writings** (Total score for memorization of each model writing is 100).

| Score deduction | Error description | Examples |
| --- | --- | --- |
| 1 point deducted for each error | One word is missing, which does not cause grammar errors. | Tourism, an indispensable part of modern business, has **(long)** been taken as being effective to boost economy. |
|  | One spelling mistake | Tourism, an **indispensble** part of modern business, has long been taken as being effective to boost economy. |
|  | One word that violates the capitalization rules | Tourism, an indispensable part of modern business, has long been taken as being effective to boost economy. **however**, a number of environmentalists contend that... |
|  | One plural form error | Constant **effort(s)** should be made … |
| 3 points deducted for each error | One word is missing, which causes grammar errors. | Tourism, an indispensable part of modern business, has long **(been)** taken as being effective to boost economy. |
|  | One passive voice error | Among the various downsides, what concerns people most is that the ecological system could be **damage** if too many business activities in regard to tourism are conducted. |
|  | One subject-verb agreement error | Among the various downsides, what concerns people most **are** that the ecological system could be damaged if too many business activities in regard to tourism are conducted. |
|  | Other possible minor grammar errors in one sentence | |
| 5 points deducted for each error | Part of a sentence is missing. | With the destruction of the environment the number of the new deceases is in an increase, so **(health problems worry people a lot.)** |
|  | Part of a sentence is rearranged | Original: Therefore, in the process of being involved in the routine works assigned by the Students’ Union, one’s communication skills could be enhanced to a large extent.  **Rearranged: Therefore, done the works by the Students’ Union, one’s abilities are increased to a large extent.** |
|  | Several important words within a sentence are missing | Some **(effective)** methods could **(assist)** the adolescents to stay recovered by **(eliminating)** the pressure. |
| 10 points deducted for each error | One complete sentence is missing |  |
| 35 points deducted for each error | One passage is missing |  |

**Appendix C:**

**1. Writing task of pre-test**

Read carefully the following excerpt and then write your response in NO LESS THAN 200 WORDS, in which you should:

1) Summarize the main message of the excerpt, and then

2) Comment on **whether our brains will get lazy in a world run by intelligent machines.**

You can support yourself with information from the excerpt.

Marks will be awarded for content relevance, content sufficiency, organization and language quality. Failure to follow the above instructions may result in a loss of marks.

Write your response on ANSWER SHEET THREE.

**With intelligent machines to do the thinking, will our brains get lazy?**

Changing technology stimulates the brain and increases intelligence. But that may only be true if the technology challenges us. In a world run by intelligent machines, our lives could get a lot simpler. Would that make us less intelligent?

Artificial intelligence is taking over many human jobs. For instance, planes are being flown much of the time by automatic pilots. And the complex problem of controlling air traffic around large modern airports is also achieved by artificial intelligence that operates well beyond the capacity of mere human air traffic controllers.

Artificial intelligence is embedded in many features of modern life for the simple reason that intelligent machines can already outperform humans, including some aptitudes where there was once thought to be a human advantage, such as playing chess, and writing poetry, or even novels.

As machines get smarter, they will do more of our thinking for us and make life easier. In the future, the electronic assistant will develop to the point that it serves similar functions as a real living butler, fulfilling requests such as: “Organize a dinner party for six on Thursday, Jeeves, and invite the usual guests.”

At that point, our long struggle with challenging technologies is at an end. Like Be Wooster, we can take it easy knowing that the hard work of planning and organizing is being done by a better brain-the electronic assistant. Starved of mental effort, our brains will regress.

**2. Writing task of post-test**

Read carefully the following excerpt and then write your response in NO LESS THAN 200 WORDS, in which you should:

1) Summarize the main message of the excerpt, and then

2) Comment on **whether parents should take children out of school for holiday during term time in order to save money**.

You can support yourself with information from the excerpt.

Marks will be awarded for content relevance, content sufficiency, organization and language quality. Failure to follow the above instructions may result in a loss of marks.

Write your response on ANSWER SHEET THREE.

**Term-time holidays will be banned**

Parents are to be banned by Michael Gove, UK’s Education Secretary, from taking their children out of school to save money on holidays.

He is to abolish the right of head teachers to “authorise absense” from the classroom, which has been used to let families take term-time breaks, and will warn them they face fines for their children not being at school.

“Any time out of school has the potential to damage a child’s education,” a senior source at the Department for Education said this weekend. “That is why the government will end the distinction between authorized and unauthorized absence.”

“This is part of the government’s wider commitment to bring down truancy levels in our schools. There will also be stricter penalties for parents and schools.”

The tough measures on truancy are part of a wider attempt by Mr. Gove to make education more academically rigorous and to tackle a culture in the educational establishment which he believes has accepted “excuses for failure”.

Russell Hobby, the general secretary of the National Association of Head Teachers, said the measure would discourage parents from trying to put pressure on heads to section term time holidays. “The high cost of holidays outside of term time is still an issue but ultimately a child’s is more important than a holiday, he said.

**Appendix D: Writing scoring rubric for pre-test and post-test**

Total score is 20.

| Scores | Descriptors |
| --- | --- |
| 20-18 | 🞄 The writing effectively addresses the writing task, presenting a well-developed organizational structure with well-supported ideas.  🞄 A wide range of vocabulary and sentence structures are used fluently and flexibly to convey precise meanings. Almost no errors of vocabulary, spelling, punctuation or syntax are made.  🞄 The writing displays an adequate ability to use the language with appropriacy |
| 17-15 | 🞄 The writing adequately addresses almost all parts of the writing task, presenting a generally well-developed organizational structure with supported ideas.  🞄 A sufficient range of vocabulary and sentence structures are used to allow some flexibility and precision. Relatively few significant errors of vocabulary, spelling, punctuation or syntax are made.  🞄 The writing displays an ability to use the language with appropriacy. |
| 14-11 | 🞄 The writing adequately addresses most of the writing task, presenting an adequately developed organizational structure, though there may occasionally be a lack of relevance, clarity, consistency or support. It presents relevant main ideas but some may be inadequately developed or unclear.  🞄 An adequate range of vocabulary and a mix of simple and complex sentence forms are used, but occasional errors of vocabulary, spelling, punctuation or syntax are made, which do not reduce communication  🞄 The writing displays some ability to use the language with appropriacy. |
| 10-7 | 🞄 The writing only addresses the writing task partially, presenting an inadequate organizational structure, and there may quite often be a lack of relevance, clarity, consistency or support. Some main ideas are presented, but limited and not sufficiently developed; there may be irrelevant details.  🞄 Frequent errors of vocabulary, spelling, punctuation or syntax are made, which can cause difficulty for the readers.  🞄 The writing displays a limited ability to use the language with appropriacy. |
| 6-1 | Almost no communication |

**Appendix E: Interview Guide Questions for Participants**

1. Could you please recall your experience of memorizing the seven model writings handed out to you?

🞄 Please describe the whole process of your text memorization.

🞄 What learning strategies did you use to help you commit the model writings into memory?

2. Do you think text memorization is effective to improve your English argumentative writing? And in what aspects?
